# Low-risk susceptibility alleles in 40 human breast cancer cell lines

**DOI:** 10.1186/1471-2407-9-236

**Published:** 2009-07-16

**Authors:** Muhammad Riaz, Fons Elstrodt, Antoinette Hollestelle, Abbas Dehghan, Jan GM Klijn, Mieke Schutte

**Affiliations:** 1 Department of Medical Oncology, Josephine Nefkens Institute, Erasmus University Medical Center, Rotterdam, the Netherlands; 2 Department of Epidemiology, Erasmus University Medical Center, Rotterdam, the Netherlands

## Abstract

**Background:**

Low-risk breast cancer susceptibility alleles or SNPs confer only modest breast cancer risks ranging from just over 1.0 to1.3 fold. Yet, they are common among most populations and therefore are involved in the development of essentially all breast cancers. The mechanism by which the low-risk SNPs confer breast cancer risks is currently unclear. The breast cancer association consortium BCAC has hypothesized that the low-risk SNPs modulate expression levels of nearby located genes.

**Methods:**

Genotypes of five low-risk SNPs were determined for 40 human breast cancer cell lines, by direct sequencing of PCR-amplified genomic templates. We have analyzed expression of the four genes that are located nearby the low-risk SNPs, by using real-time RT-PCR and Human Exon microarrays.

**Results:**

The SNP genotypes and additional phenotypic data on the breast cancer cell lines are presented. We did not detect any effect of the SNP genotypes on expression levels of the nearby-located genes *MAP3K1, FGFR2, TNRC9 *and *LSP1*.

**Conclusion:**

The SNP genotypes provide a base line for functional studies in a well-characterized cohort of 40 human breast cancer cell lines. Our expression analyses suggest that a putative disease mechanism through gene expression modulation is not operative in breast cancer cell lines.

## Background

About ten percent of breast cancer patients have a history of multiple breast cancer cases in their family, suggesting the inheritance of breast cancer susceptibility alleles in these families. Germline mutations in the *BRCA1 *and *BRCA2 *genes are identified in about one quarter of the families with breast cancer. Female carriers of *BRCA1 *and *BRCA2 *mutations have an estimated 50–90% life-time risk to develop breast cancer, classifying both genes as high-risk susceptibility genes [1,2]. Other high-risk breast cancer genes include the *p53*, *PTEN *and *STK11 *genes, but mutations in these genes account for only few familial breast cancers. *CHEK2 *was the first moderate-risk breast cancer gene being identified [3-5]. Germline mutations in *CHEK2 *are identified in up to 5% of breast cancer families, albeit that their prevalence varies widely among populations. Female carriers of *CHEK2 *mutations have a moderate two to three fold increased risk to develop breast cancer. By now, several other moderate-risk breast cancer genes have been identified, including *ATM*, *BRIP1 *and *PALB2 *[6-9]. Mutations in these genes all confer increased breast cancer risks of two to three fold and mutations in each of these genes are identified in about 1% of the familial breast cancers. Recently, the international breast cancer association consortium (BCAC) has conducted a large genome-wide association study and identified five single nucleotide polymorphisms (SNPs) that associated with breast cancer [10]. Four of these SNPs were within haplotype blocks that contained genes: SNP rs2981582 locates in intron 2 of the *FGFR2 *gene at chromosome 10q; SNP rs889312 locates near *MAP3K1 *at 5q; SNP rs3803662 locates between *TNRC9 *and the LOC643714 gene at 16q; and SNP rs3817198 locates intronic in *LSP1 *at 11p. SNP rs13281615 locates at 8q24 in a region without any annotated genes. Importantly, independent genome-wide association studies have associated other SNPs in *FGFR2 *with breast cancer [11,12]. As *FGFR2 *had already been implicated in breast cancer [13-20], the significance of the *FGFR2 *SNPs as susceptibility alleles seemed evident. The *TNRC9 *SNP had also been associated with breast cancer in another study [21]. Lastly, the 8q24 SNP was of particular interest because other SNPs at 8q24 had been associated with increased risks of prostate cancer and colorectal cancer [22-26]. BCAC estimated that each of the five identified SNPs associated with rather small increased breast cancer risks, ranging from just over 1.0 to 1.3 fold, classifying them as low-risk susceptibility alleles [10]. However, these low-risk SNPs are very common and their impact is therefore still substantial, together accounting for almost 5% of the familial breast cancers.

The mechanism by which the low-risk susceptibility alleles confer breast cancer risks was obscure [10]. In analogy with the high-risk and moderate-risk breast cancer genes, it had been anticipated that the identified SNPs associated with disease-causing alleles in the coding sequences of nearby located genes. However, extensive sequencing efforts have not identified such alleles in the SNP-associated haplotype blocks, suggesting that the SNPs themselves might be the disease-causing susceptibility alleles [10]. BCAC therefore proposed an alternative disease mechanism that involves expression modulation of genes located in the vicinity of the identified SNPs, thereby conferring low breast cancer risks. Here, we have evaluated expression modulation in a well-characterized cohort of 40 human breast cancer cell lines, allowing us to specifically address whether this mechanism might operate in breast cancer cells.

## Methods

### Breast cancer cell lines

The 40 human breast cancer cell lines used in this study are listed in Table 1 and have been described in detail elsewhere [27]. Microsatellite analysis with nearly 150 polymorphic markers had shown that all cell lines are unique and monoclonal [28].

**Table 1:** Genotypes of five low-risk SNPs in 40 human breast cancer cell lines

Breast cancer cell lines	SNP genotypes and allelic losses									
	
	8q24	Loss 8q	*MAP3K1*	Loss 5q	*FGFR2*	Loss 10q	*TNRC9*	Loss 16q	*LSP1*	Loss 11p
SUM185PE	Het	No	Het	No	Maj H	nd	Min H	nd	Maj H	nd
BT483	Het	No	Maj H	No	Min H	Yes	Maj H	No	Maj H	No
MDA-MB-134VI	Het	No	Het	No	Het	No	Het	No	Maj H	No
MDA-MB-175VII	Het	No	Min H	No	Het	No	Het	No	Maj H	No
MDA-MB-415	Het	No	Het	No	Min H	Yes	Het	No	Het	No
MPE600	Het	No	Maj H	nd	Het	No	Maj H	nd	Het	No
SUM52PE	Maj H	nd	Maj H	nd	Maj H	nd	Maj H	nd	Maj H	nd
CAMA-1	Het	No	Het	No	Min H	No	Maj H	Yes	Maj H	Yes
MCF-7	Maj H	No	Het	No	Maj H	No	Het	No	Maj H	Yes
ZR75-1	Het	No	Het	No	Min H	Yes	Maj H	Yes	Het	No
SUM44PE	Het	No	Min H	nd	Maj H	nd	Het	No	Min H	nd
T47D	Het	No	Het	No	Maj H	No	Min H	Yes	Min H	Yes
MDA-MB-361	Het	No	Het	No	Maj H	No	Het	No	Min H	No
BT474	Het	No	Maj H	No	Maj H	Yes	Maj H	No	Maj H	No
UACC812	Min H	No	Min H	No	Het	No	Het	No	Min H	Yes
ZR75-30	Maj H	nd	Min H	nd	Min H	nd	Min H	nd	Maj H	nd
OCUB-F	Maj H	nd	Maj H	nd	Min H	nd	Min H	nd	Maj H	nd
SK-BR-5	Het	No	Maj H	nd	Min H	nd	Min H	nd	Maj H	nd
SUM190PT	Min H	nd	Min H	nd	Maj H	nd	Min H	nd	Maj H	nd
SUM225CWN	Het	No	Maj H	nd	Maj H	nd	Maj H	nd	Het	No
MDA-MB-330	Het	No	Min H	Yes	Min H	Yes	Het	No	Maj H	No
MDA-MB-453	Het	No	Het	No	Min H	Yes	Maj H	Yes	Maj H	Yes
SK-BR-3	Min H	Yes	Het	No	Het	No	Maj H	Yes	Min H	No
EVSA-T	Min H	nd	Min H	nd	Maj H	nd	Maj H	nd	Min H	nd
UACC893	Maj H	No	Maj H	No	Maj H	Yes	Het	No	Maj H	No
BT20	Min H	No	Maj H	Yes	Maj H	Yes	Maj H	Yes	Het	No
HCC1937	Het	No	Min H	nd	Het	No	Het	No	Maj H	nd
MDA-MB-468	Maj H	Yes	Maj H	Yes	Maj H	Yes	Maj H	Yes	Maj H	Yes
SUM149PT	Min H	nd	Het	No	Min H	nd	Min H	nd	Maj H	Yes
SUM229PE	Maj H	nd	Maj H	nd	Maj H	nd	Maj H	nd	Min H	nd
BT549	Maj H	No	Maj H	No	Maj H	Yes	Het	No	Min H	Yes
Hs578T	Het	No	Maj H	Yes	Min H	Yes	Min H	No	Maj H	No
MDA-MB-157	Maj H	No	Maj H	Yes	Maj H	Yes	Min H	No	Maj H	Yes
MDA-MB-231	Maj H	Yes	Het	No	Het	No	Maj H	Yes	Het	No
MDA-MB-436	Maj H	Yes	Maj H	Yes	Maj H	No	Min H	No	Maj H	Yes
SK-BR-7	Het	No	Het	No	Min H	nd	Maj H	nd	Het	No
SUM159PT	Maj H	nd	Het	No	Min H	nd	Het	No	Maj H	nd
SUM1315MO2	Het	No	Maj H	nd	Min H	nd	Het	No	Maj H	nd
SUM102PT	Het	No	Het	No	Maj H	nd	Het	No	Maj H	nd
MDA-MB-435s	Maj H	No	Maj H	Yes	Maj H	Yes	Maj H	Yes	Maj H	No
										
*Total major homozygotes*	13		17		19		16		25	
*Total heterozygotes*	21		15		7		14		7	
*Total minor homozygotes*	6		8		14		10		8	
*Percentage of allelic loss*		13		25		52		32		37

Genotypes of five low-risk SNPs have been determined in the current study. Allelotype data have been reported elsewhere and involved microsatellite analysis. Loss: Yes, allelic loss at the indicated chromosomal region; and No, no allelic loss at the indicated chromosomal region. nd, not determined; Maj H, major homozygotes; Min H, minor homozygotes; Het, heterozygote allele carriers.

### Genotyping

Genotypes of five low-risk susceptibility alleles have been determined: rs889312 (A>C) near the *MAP3K1 *gene; rs2981582 (C>T) in the *FGFR2 *gene; rs3803662 (C>T) near the *TNRC9 *gene; rs3817198 (T>C) in the *LSP1 *gene and rs13281615 (A>G) that located in a gene desert at chromosome 8q24 [10]. Genotyping was performed by direct sequencing of PCR-amplified genomic templates, using the BigDye Terminator V3.1 Cycle Sequencing Kit (Applied Biosystems) and an ABI 3130xL Genetic Analyzer. Primer sequences are available upon request.

Allele frequencies of cases and controls reported by BCAC have been obtained by using their reported Odds Ratio data [10], and inferring allele frequencies by assuming that Odds Ratios reflect the ratio of minor allele carriers versus major allele carriers from the cases divided by the ratio of minor allele carriers versus major allele carriers from the controls.

### Expression analysis

Transcript expression levels of four genes have been determined: *MAP3K1*, *FGFR2*, *TNRC9 *and *LSP1*. Quantitative real-time PCR (qPCR) was performed on cDNA templates that had been generated with oligo-dT and random hexamer primers from total RNA isolates, using *Power *SYBR Green PCR Master Mix (Applied Biosystems) and an ABI Prism 7700. Ct values were normalized according *HPRT *and *HMBS *housekeeper Ct values. Transcript expression had also been determined by Human Exon 1.0 ST microarrays (Affymetrix), as described elsewhere [29]. The exon array data have been deposited in NCBI's Gene Expression Omnibus [30] and are accessible through GEO Series accession number GSE16732.

### Statistical analysis

Statistical analyses were performed with Statistical Package for the Social Sciences (SPSS) version 11.5, considering P-values of less than 0.05 significant. Fisher's exact test was used to determine association of the SNP genotypes with the breast cancer cell lines. The Kruskal Wallis test was used to compare gene expression levels among three SNP genotype groups (major homozygotes, heterozygotes, and minor homozygotes).

## Results and discussion

### Genotyping of low-risk susceptibility alleles in breast cancer cell lines

**Figure 1:**
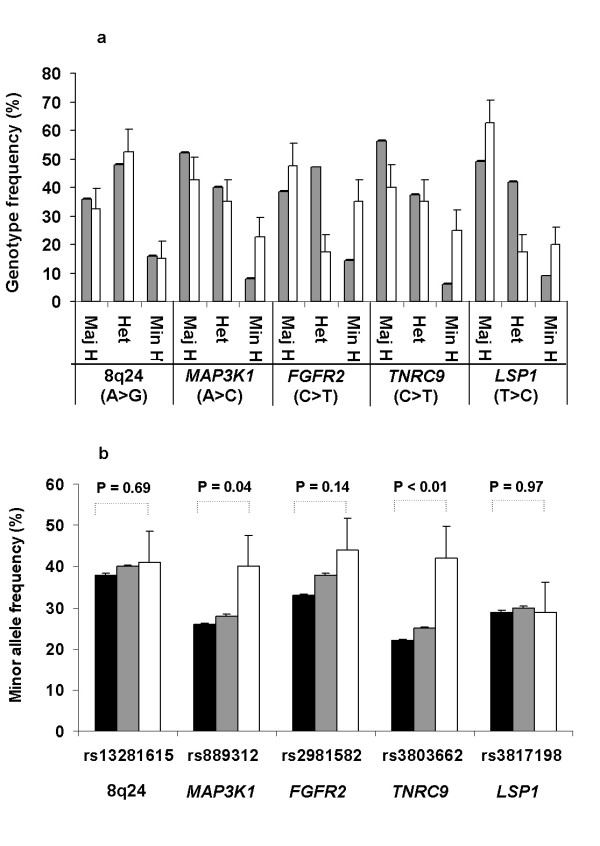
**Genotypes and minor allele frequencies of five low-risk breast cancer susceptibility alleles or SNPs in human breast cancer cell lines**. **1a**. Gray bars represent SNP genotype frequencies of 21,860 blood-derived samples from breast cancer cases reported by the breast cancer association consortium BCAC [10], and white bars represent genotype frequencies in 40 breast cancer cell lines. Maj H, major homozygotes; Min H, minor homozygotes; and Het, heterozygote allele carriers. The major and minor alleles of each allele are indicated between brackets. **1b**. Black and gray bars represent minor allele frequencies in 22,578 population controls and 21,860 breast cancer cases, respectively, as reported by BCAC [10]. White bars represent frequencies identified in 40 breast cancer cell lines.

Genotypes of five low-risk susceptibility alleles [10] were determined in a cohort of 40 human breast cancer cell lines. For each SNP, frequencies of major homozygotes, heterozygotes and minor homozygotes are shown in Figure 1a and genotypes are detailed in Table 1. Frequencies of homozygote genotypes typically were higher than anticipated, likely related to allelic losses in the cell line samples (Figure 1a; [10]). For four SNPs (*8q24*, *MAP3K1*, *FGFR2 *and *TNRC9*), the minor allele frequencies among the cell lines were higher than among the 21,860 BCAC breast cancer cases and 22,578 population controls (Figure 1b; [10]). Fisher's exact testing indicated that the minor allele frequencies among the cell lines were significantly higher than the BCAC population controls for two SNPs: *MAP3K1 *and *TNRC9 *(Figure 1b). In Table 1 and 2, we also included previously-determined phenotypic and genotypic data on the breast cancer cell lines, including data on molecular subtyping and allelotyping (Hollestelle *et al*. submitted for publication; [28]). Together with the SNP genotypes, we provide a base line for functional studies in this cohort of breast cancer cell lines.

**Table 2:** Molecular and phenotypic characterizations of 40 breast cancer cell lines

Breast cancer cell lines	Breast cancer subtype	Intrinsic subtype	Protein expression			
			
			ER	PgR	ERBB2	3-neg
SUM185PE	Luminal-type	Luminal	-	-	-	+
BT483	Luminal-type	Luminal	+	-	+	-
MDA-MB-134VI	Luminal-type	Luminal	+	-	-	-
MDA-MB-175VII	Luminal-type	Luminal	+	-	-	-
MDA-MB-415	Luminal-type	Luminal	+	-	-	-
MPE600	Luminal-type	Luminal	+	-	+	-
SUM52PE	Lumina-type	Luminal	+	-	+	-
CAMA-1	Lumina-type	Luminal	+	+	+	-
MCF-7	Luminal-type	Luminal	+	+	-	-
ZR75-1	Luminal-type	Luminal	+	+	+	-
SUM44PE	Luminal-type	Luminal	+	+	-	-
T47D	Luminal-type	Luminal	+	+	-	-
MDA-MB-361	Luminal-type	Luminal	+	+	++	-
BT474	Luminal-type	Luminal	-	+	++	-
UACC812	Luminal-type	Luminal	-	+	++	-
ZR75-30	Luminal-type	Luminal	+	-	++	-
OCUB-F	Luminal-type	Luminal	-	-	++	-
SK-BR-5	Luminal-type	Luminal	-	-	++	-
SUM190PT	Luminal-type	nd	-	-	++	-
SUM225CWN	Luminal-type	nd	-	-	++	-
MDA-MB-330	Luminal-type	ERBB2	+	-	++	-
MDA-MB-453	Luminal-type	ERBB2	-	-	++	-
SK-BR-3	Luminal-type	ERBB2	-	-	++	-
EVSA-T	Luminal-type	ERBB2	-	-	++	-
UACC893	Luminal-type	ERBB2	-	-	++	-
BT20	Basal-type	Basal-like	-	-	-	+
HCC1937	Basal-type	Basal-like	-	-	-	+
MDA-MB-468	Basal-type	Basal-like	-	-	-	+
SUM149PT	Basal-type	Basal-like	-	-	-	+
SUM229PE	Basal-type	Basal-like	-	-	-	+
BT549	Basal-type	Normal-like	-	-	-	+
Hs578T	Basal-type	Normal-like	-	-	-	+
MDA-MB-157	Basal-type	Normal-like	-	-	-	+
MDA-MB-231	Basal-type	Normal-like	-	-	-	+
MDA-MB-436	Basal-type	Normal-like	-	-	-	+
SK-BR-7	Basal-type	Normal-like	-	-	-	+
SUM159PT	Basal-type	Normal-like	-	-	-	+
SUM1315MO2	Basal-type	Normal-like	-	-	-	+
SUM102PT	Basal-type	Normal-like	nd	nd	nd	nd
MDA-MB-435s	Basal-type	Normal-like	-	-	-	+
						
*Total phenotype positives*			14	8	13	16

Phenotypic characterizations have been reported elsewhere and involved protein expression patterns of the cell lines for the breast cancer subtyping (cytokeratins, ER, PgR and ERBB2) and expression of the intrinsic gene set for the intrinsic subtyping (Hollestelle *et al*. submitted for publication). Protein expression: +, expressed; ++, over expressed; and -, not detectable; 3-neg, triple-negative.

### Expression levels of nearby located genes in breast cancer cell lines do not correlate with their SNP genotype

Surprisingly, BCAC had not identified disease-causing gene variants within the haplotype blocks of the five low-risk SNPs [10]. They proposed an alternative disease mechanism, in which SNP genotypes modulate expression levels of nearby located genes. Such disease mechanism was conceivable because the minor SNP alleles confer only low risks for breast cancer. Here, we have evaluated whether gene expression modulation is operative in breast cancer cell lines, by associating SNP genotypes of the breast cancer cell lines with the expression levels of nearby located genes.

**Figure 2:**
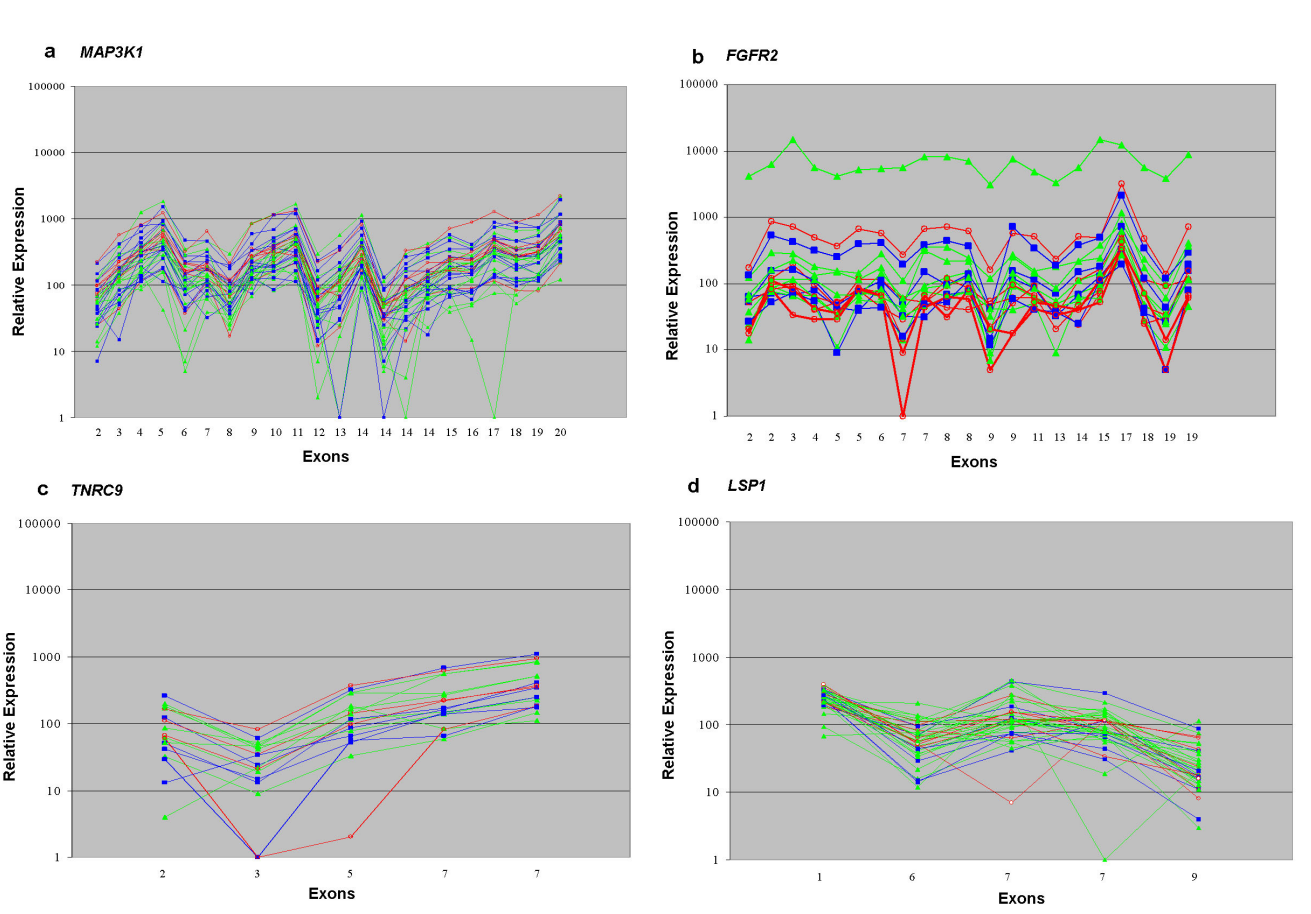
**Normalized expression levels from Affymetrix Human Exon 1.0 ST microarrays of 2a. *MAP3K1*; 2b. *FGFR2*; 2c. *TNRC9*; and 2d. *LSP1*; from 40 human breast cancer cell lines**. Kruskal Wallis testing using the average expression among all probe sets for each gene did not reveal significant associations between gene expression and SNP genotypes. Each line represents a cell line, with the color-coding according the genotype groups: green, major homozygotes; red, minor homozygotes; and blue, heterozygotes. Two cell lines with the delEx9 isoform of *FGFR2 *are indicated with bold red lines. Probe sets for each gene were ordered by physical location and indicated by exon, where probe sets that were not unique for that gene were omitted. Probe sets with expression values less than the background of 50 were also omitted, unless more than 3 cell lines had expression levels higher than 100.

**Figure 3:**
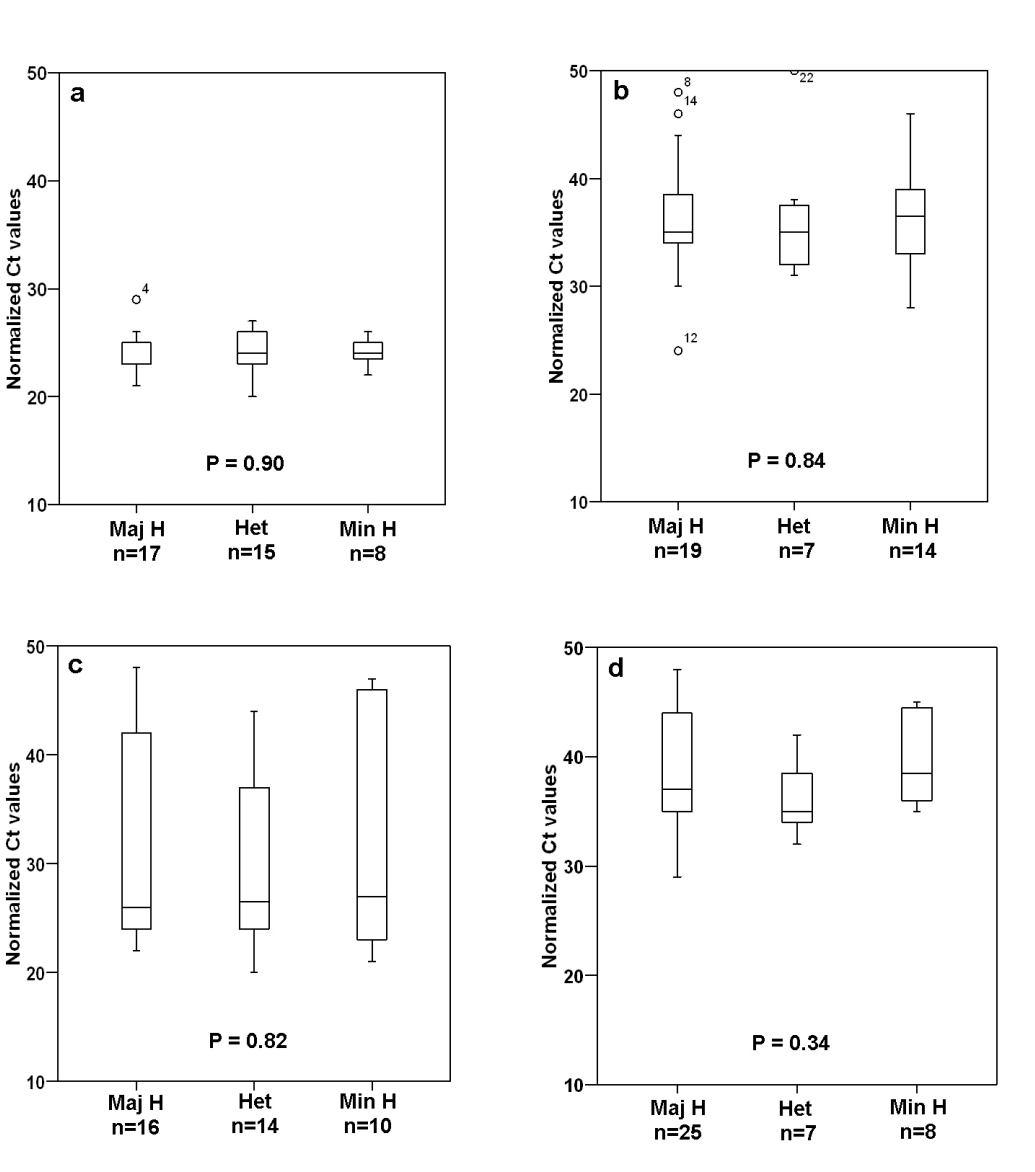
**Correlation of gene expression levels of 3a. *MAP3K1*; 3b. *FGFR2*; 3c. *TNRC9*; and 3d. *LSP1*; with the SNP genotypes in 40 human breast cancer cell lines**. Kruskal Wallis testing did not reveal any significant association between gene expression and SNP genotypes. Maj H, major homozygotes; Min H, minor homozygotes; and Het, heterozygote allele carriers. The number of cell lines in each genotype group is indicated under the genotypes and data are detailed in Table 1.

Gene expression data of the four genes physically nearest to the SNPs were obtained by Affymetrix Human Exon 1.0 ST microarray profiling and by qPCR analysis. Both transcript expression analysis methods revealed similar expression levels for each of the four genes: *MAP3K1*, *FGFR2*, *TNRC9 *and *LSP1*, with Spearman correlation coefficients of -0.6, -0.7, -0.8 and -0.4, respectively, among the 40 breast cancer cell lines (Table 3 and Figures 2 and 3). Because BCAC had shown that the low-risk SNPs confer breast cancer risks in a dose-dependent manner, with the highest risks for the minor homozygotes [10], association between gene expression levels and SNP genotypes was performed by three-group comparisons. Exon array data are shown in Figure 2, with cell lines from each genotype group depicted in a different color. Unique outliers typically represented decreased expression of one or more probes sets, such as exon 17 of *MAP3K1 *or exons 3–5 of *TNRC9*, possibly related to the presence of SNPs in probe sequences, alternative splicing or genomic deletions [29]. Expression of recurrent isoforms as reported by NCBI was detected only for the *FGFR2 *gene, with two cell lines expressing the isoform that lacked exon 9. Both cell lines were minor homozygotes for the *FGFR2 *SNP. Overall, there was no apparent association between the exon array expression level of each of the four genes and their SNP genotypes (Figure 2). The qPCR Ct-values are detailed in Table 3 and the three-group comparisons are shown in Figure 3. Again, we did not detect any association between gene expression levels with SNP genotypes for the four genes. It is possible that gene expression levels are affected by allelic loss of the gene loci. We therefore also have compared gene expression levels in major and minor homozygotes with allelic loss to the gene expression levels in cell lines without allelic loss, but gene expression levels did not correlate with allelic losses either (Table 4). Altogether, these results strongly suggest that a putative disease mechanism by expression modulation does not operate via cancer cells. Yet, recent studies have shown that expression levels of the *FGFR2*, *MAP3K1 *and *TNRC9 *genes associated with their SNP genotype in clinical breast cancer samples [31,32]. It may be that expression modulation is operative in non-neoplastic stromal or epithelial cells and perhaps only early in carcinogenesis. Alternatively, it may be that expression modulation of these genes was operative in invasive breast cancer cells but was lost upon *in vitro *propagation of the cell lines. Expression analysis of carefully dissected tumor cells and non-neoplastic epithelial and stromal cells from clinical breast cancer samples should resolve this issue and may determine the precise mechanism of expression modulation by low-risk breast cancer susceptibility alleles.

**Table 3:** Gene expression analysis of *MAP3K1, FGFR2*, *TNRC9 *and *LSP1 *in 40 human breast cancer cell lines by quantitative RT-PCR, represented by normalized Ct values

Breast cancer cell lines	Transcript expression (normalized Ct values)			
	
	*MAP3K1*	*FGFR2*	*TNRC9*	*LSP1*
BT20	23	30	45	38
BT474	24	35	22	35
BT483	22	31	25	36
BT549	29	32	39	40
CAMA-1	24	33	26	45
EVSA-T	25	33	25	45
HCC1937	26	32	37	45
Hs578T	26	45	45	34
MCF-7	25	34	28	39
MDA-MB-134VI	22	37	24	35
MDA-MB-157	25	44	33	29
MDA-MB-175VII	24	35	23	36
MDA-MB-231	26	45	45	35
MDA-MB-330	26	32	27	33
MDA-MB-361	23	34	23	44
MDA-MB-415	23	28	20	33
MDA-MB-435s	25	45	45	45
MDA-MB-436	26	35	31	37
MDA-MB-453	24	36	29	45
MDA-MB-468	25	35	37	37
MPE600	23	31	22	42
OCUB-F	23	39	23	42
SK-BR-3	25	32	26	37
SK-BR-5	23	37	21	43
SK-BR-7	26	39	45	35
SUM102PT	25	35	32	29
SUM1315M02	26	43	44	35
SUM149PT	27	38	45	37
SUM159PT	27	45	43	34
SUM185PE	23	38	23	45
SUM190PT	24	45	23	45
SUM225CWN	24	36	23	39
SUM229PE	25	39	33	36
SUM44PE	22	41	26	36
SUM52PE	24	24	24	37
T47D	20	36	45	35
UACC812	24	38	24	45
UACC893	21	36	25	36
ZR75-1	24	36	24	32
ZR75-30	23	33	23	43
				
*Total high expressers (Ct <*20)	1	0	1	0
*Total moderate expressers (Ct 20–30)*	39	2	23	2
*Total low expressers (Ct >*30–35)	0	12	4	5
*Total no expressers (Ct >*35)	0	26	12	33

**Table 4:** Gene expression of *MAP3K1*, *FGFR2*, *TNRC9 *and *LSP1 *in human breast cancer cell lines according to their allelic loss status at the gene locus

Gene	Genotype (Number of cell lines)	Average expression level (Normalized Ct values)
*MAP3K1*	Het (n = 14)	24 ± 2
	Maj H no loss (n = 4)	24 ± 4
	Min H no loss (n = 2)	24 ± 0
	Maj H allelic loss (n = 1)	26
	Min H allelic loss (n = 6)	25 ± 1

*FGFR2*	Het (n = 7)	36 ± 5
	Maj H no loss (n = 4)	31 ± 7
	Min H no loss (n = 1)	33
	Maj H allelic loss (n = 6)	35 ± 6
	Min H allelic loss (n = 6)	38 ± 6

*TNRC9*	Het (n = 14)	30 ± 8
	Maj H no loss (n = 2)	24 ± 2
	Min H no loss (n = 3)	36 ± 8
	Maj H allelic loss (n = 1)	45
	Min H allelic loss (n = 7)	36 ± 9

*LSP1*	Het (n = 7)	37 ± 4
	Maj H no loss (n = 8)	37 ± 4
	Min H no loss (n = 2)	41 ± 5
	Maj H allelic loss (n = 3)	40 ± 5
	Min H allelic loss (n = 7)	38 ± 6

Although numbers are small for some sample groups, there are no apparent differences in gene expression levels related to allelic loss status. Allelic loss data and qPCR expression data are detailed in Table 1 and Table 3, respectively. Maj H, major homozygotes; Min H, minor homozygotes; Het, heterozygote allele carriers.

## Conclusion

We present the genotypes of five low-risk susceptibility alleles or SNPs of 40 human breast cancer cell lines. Using this cell line model, we have evaluated the BCAC hypothesis that low-risk SNPs confer breast cancer risks by modulation of expression levels of nearby located genes. We found no evidence for expression modulation in the breast cancer cell lines, suggesting that such disease mechanism is more likely to operate in non-neoplastic epithelial or stromal cells or has been lost during *in vitro *propagation of the cell lines.

## Competing interests

The authors declare that they have no competing interests.

## Authors' contributions

MR and FE carried out genotyping and transcript expression analyses, AH carried out protein expression analyses, and MR and AD performed statistical analyses. MR, JGMK and MS designed the study, and MR and MS wrote the manuscript. All authors read and approved the final manuscript.

## Pre-publication history

The pre-publication history for this paper can be accessed here:

http://www.biomedcentral.com/1471-2407/9/236/prepub

## References

[B1] Thompson D Easton D The genetic epidemiology of breast cancer genes J Mammary Gland Biol Neoplasia 2004 9 3 221 236 10.1023/B:JOMG.0000048770.90334.3b 15557796

[B2] Walsh T King MC Ten genes for inherited breast cancer Cancer Cell 2007 11 2 103 105 10.1016/j.ccr.2007.01.010 17292821

[B3] Meijers-Heijboer H Ouweland A van den Klijn J Wasielewski M de Snoo A Oldenburg R Hollestelle A Houben M Crepin E van Veghel-Plandsoen M Low-penetrance susceptibility to breast cancer due to CHEK2(*)1100delC in noncarriers of BRCA1 or BRCA2 mutations Nat Genet 2002 31 1 55 59 10.1038/ng879 11967536

[B4] Vahteristo P Bartkova J Eerola H Syrjakoski K Ojala S Kilpivaara O Tamminen A Kononen J Aittomaki K Heikkila P A CHEK2 genetic variant contributing to a substantial fraction of familial breast cancer Am J Hum Genet 2002 71 2 432 438 12094328 10.1086/341943 PMC379177

[B5] Consortium CBCC-C CHEK2*1100delC and susceptibility to breast cancer: a collaborative analysis involving 10,860 breast cancer cases and 9,065 controls from 10 studies Am J Hum Genet 2004 74 6 1175 1182 15122511 10.1086/421251 PMC1182081

[B6] Renwick A Thompson D Seal S Kelly P Chagtai T Ahmed M North B Jayatilake H Barfoot R Spanova K ATM mutations that cause ataxia-telangiectasia are breast cancer susceptibility alleles Nat Genet 2006 38 8 873 875 10.1038/ng1837 16832357

[B7] Seal S Thompson D Renwick A Elliott A Kelly P Barfoot R Chagtai T Jayatilake H Ahmed M Spanova K Truncating mutations in the Fanconi anemia J gene BRIP1 are low-penetrance breast cancer susceptibility alleles Nat Genet 2006 38 11 1239 1241 10.1038/ng1902 17033622

[B8] Rahman N Seal S Thompson D Kelly P Renwick A Elliott A Reid S Spanova K Barfoot R Chagtai T PALB2, which encodes a BRCA2-interacting protein, is a breast cancer susceptibility gene Nat Genet 2007 39 2 165 167 10.1038/ng1959 17200668 PMC2871593

[B9] Stratton MR Rahman N The emerging landscape of breast cancer susceptibility Nat Genet 2008 40 1 17 22 10.1038/ng.2007.53 18163131

[B10] Easton DF Pooley KA Dunning AM Pharoah PD Thompson D Ballinger DG Struewing JP Morrison J Field H Luben R Genome-wide association study identifies novel breast cancer susceptibility loci Nature 2007 447 7148 1087 1093 10.1038/nature05887 17529967 PMC2714974

[B11] Hunter DJ Kraft P Jacobs KB Cox DG Yeager M Hankinson SE Wacholder S Wang Z Welch R Hutchinson A A genome-wide association study identifies alleles in FGFR2 associated with risk of sporadic postmenopausal breast cancer Nat Genet 2007 39 7 870 874 10.1038/ng2075 17529973 PMC3493132

[B12] Gold B Kirchhoff T Stefanov S Lautenberger J Viale A Garber J Friedman E Narod S Olshen AB Gregersen P Genome-wide association study provides evidence for a breast cancer risk locus at 6q22.33 Proc Natl Acad Sci USA 2008 105 11 4340 4345 18326623 10.1073/pnas.0800441105 PMC2393811

[B13] Adnane J Gaudray P Dionne CA Crumley G Jaye M Schlessinger J Jeanteur P Birnbaum D Theillet C BEK and FLG, two receptors to members of the FGF family, are amplified in subsets of human breast cancers Oncogene 1991 6 4 659 663 1851551

[B14] Luqmani YA Graham M Coombes RC Expression of basic fibroblast growth factor, FGFR1 and FGFR2 in normal and malignant human breast, and comparison with other normal tissues Br J Cancer 1992 66 2 273 280 1380281 10.1038/bjc.1992.256 PMC1977809

[B15] Penault-Llorca F Bertucci F Adelaide J Parc P Coulier F Jacquemier J Birnbaum D deLapeyriere O Expression of FGF and FGF receptor genes in human breast cancer Int J Cancer 1995 61 2 170 176 10.1002/ijc.2910610205 7705943

[B16] Dickson C Spencer-Dene B Dillon C Fantl V Tyrosine kinase signalling in breast cancer: fibroblast growth factors and their receptors Breast Cancer Res 2000 2 3 191 196 11250709 10.1186/bcr53 PMC138774

[B17] Heiskanen M Kononen J Barlund M Torhorst J Sauter G Kallioniemi A Kallioniemi O CGH, cDNA and tissue microarray analyses implicate FGFR2 amplification in a small subset of breast tumors Anal Cell Pathol 2001 22 4 229 234 11564899 10.1155/2001/981218 PMC4615989

[B18] Antoniou A Pharoah PD Narod S Risch HA Eyfjord JE Hopper JL Loman N Olsson H Johannsson O Borg A Average risks of breast and ovarian cancer associated with BRCA1 or BRCA2 mutations detected in case Series unselected for family history: a combined analysis of 22 studies Am J Hum Genet 2003 72 5 1117 1130 12677558 10.1086/375033 PMC1180265

[B19] Moffa AB Tannheimer SL Ethier SP Transforming potential of alternatively spliced variants of fibroblast growth factor receptor 2 in human mammary epithelial cells Mol Cancer Res 2004 2 11 643 652 15561780

[B20] Greenman C Stephens P Smith R Dalgliesh GL Hunter C Bignell G Davies H Teague J Butler A Stevens C Patterns of somatic mutation in human cancer genomes Nature 2007 446 7132 153 158 17344846 10.1038/nature05610 PMC2712719

[B21] Stacey SN Manolescu A Sulem P Rafnar T Gudmundsson J Gudjonsson SA Masson G Jakobsdottir M Thorlacius S Helgason A Common variants on chromosomes 2q35 and 16q12 confer susceptibility to estrogen receptor-positive breast cancer Nat Genet 2007 39 7 865 869 10.1038/ng2064 17529974

[B22] Gudmundsson J Sulem P Manolescu A Amundadottir LT Gudbjartsson D Helgason A Rafnar T Bergthorsson JT Agnarsson BA Baker A Genome-wide association study identifies a second prostate cancer susceptibility variant at 8q24 Nat Genet 2007 39 5 631 637 10.1038/ng1999 17401366

[B23] Haiman CA Patterson N Freedman ML Myers SR Pike MC Waliszewska A Neubauer J Tandon A Schirmer C McDonald GJ Multiple regions within 8q24 independently affect risk for prostate cancer Nat Genet 2007 39 5 638 644 17401364 10.1038/ng2015 PMC2638766

[B24] Haiman CA Le Marchand L Yamamato J Stram DO Sheng X Kolonel LN Wu AH Reich D Henderson BE A common genetic risk factor for colorectal and prostate cancer Nat Genet 2007 39 8 954 956 17618282 10.1038/ng2098 PMC2391283

[B25] Tomlinson I Webb E Carvajal-Carmona L Broderick P Kemp Z Spain S Penegar S Chandler I Gorman M Wood W A genome-wide association scan of tag SNPs identifies a susceptibility variant for colorectal cancer at 8q24.21 Nat Genet 2007 39 8 984 988 10.1038/ng2085 17618284

[B26] Zanke BW Greenwood CM Rangrej J Kustra R Tenesa A Farrington SM Prendergast J Olschwang S Chiang T Crowdy E Genome-wide association scan identifies a colorectal cancer susceptibility locus on chromosome 8q24 Nat Genet 2007 39 8 989 994 10.1038/ng2089 17618283

[B27] Elstrodt F Hollestelle A Nagel JH Gorin M Wasielewski M Ouweland A van den Merajver SD Ethier SP Schutte M BRCA1 mutation analysis of 41 human breast cancer cell lines reveals three new deleterious mutants Cancer Res 2006 66 1 41 45 10.1158/0008-5472.CAN-05-2853 16397213

[B28] Harkes IC Elstrodt F Dinjens WN Molier M Klijn JG Berns EM Schutte M Allelotype of 28 human breast cancer cell lines and xenografts Br J Cancer 2003 89 12 2289 2292 10.1038/sj.bjc.6601448 14676808 PMC2395277

[B29] Schutte M Elstrodt F Bralten LB Nagel JH Duijm E Hollestelle A Vuerhard MJ Wasielewski M Peeters JK Spek P van der Exon expression arrays as a tool to identify new cancer genes PLoS ONE 2008 3 8 e3007 10.1371/journal.pone.0003007 PMC2500185 18688287

[B30] Edgar R Domrachev M Lash AE Gene Expression Omnibus: NCBI gene expression and hybridization array data repository Nucleic Acids Res 2002 30 1 207 210 11752295 10.1093/nar/30.1.207 PMC99122

[B31] Nordgard SH Johansen FE Alnaes GI Naume B Borresen-Dale AL Kristensen VN Genes harbouring susceptibility SNPs are differentially expressed in the breast cancer subtypes Breast Cancer Res 2007 9 6 113 18036273 10.1186/bcr1784 PMC2246167

[B32] Meyer KB Maia AT O'Reilly M Teschendorff AE Chin SF Caldas C Ponder BA Allele-specific up-regulation of FGFR2 increases susceptibility to breast cancer PLoS Biol 2008 6 5 e108 18462018 10.1371/journal.pbio.0060108 PMC2365982

